# Green synthesis of silver nanoparticles from *Cuscuta epithymum* extract, evaluation of antibacterial, antioxidant activity, cytotoxic effect on MCF-7 cell line

**DOI:** 10.1016/j.mex.2025.103363

**Published:** 2025-05-10

**Authors:** M. Fereydani, A. Jalalian, N. Saber

**Affiliations:** a Department of Chemistry, Faculty of Chemistry, Mazandaran University, Babolsar, Iran; b Department of Pharmacology and Toxicology, and Pharmaceutical Sciences Research Center, Pharmaceutical Sciences Branch, Islamic Azad University, Tehran, Iran; c Department of Chemistry, Faculty of Convergent Science and Technology, Science and Research Branch, Islamic Azad University, Tehran, Iran

**Keywords:** Antioxidant, Breast cancer, Cuscuta epithymum, Silver nanoparticles, Green synthesis, Green synthesis of silver nanoparticles, evaluation of antibacterial, antioxidant activity, cytotoxic effect on MCF-7 cell line

## Abstract

Using plants for the green synthesis of nanoparticles is a cost-effective, non-toxic, and environmentally friendly method. This study synthesized silver nanoparticles using *Cuscuta epithymum* extract, and their biological activities were evaluated. The *C. epithymum* extract was prepared by maceration and the synthesis of AgNPs using a green method. Confirmation of AgNPs formation was achieved through UV-Vis and the absorption peak was observed at 425 nm, and their morphology and functional groups were determined by FESEM, TEM, XRD, and FT-IR. The nanoparticles were spherical with a size of 15–60 nm. The antioxidant activity of AgNPs was calculated using the DPPH assay (IC_50_=45.55 mg/L), and antibacterial properties were obtained by Disk Diffusion methods showed the AgNPs had strong antimicrobial activity. MTT assay showed that the AgNPs caused cytotoxicity in MCF-7 with an IC_50_=42.53 mg/L, 36.78 mg/L, and 26.86 mg/L (*P* < 0.0001) after 12, 24, and 48 respectively. It can be concluded that *C. epithymum* extract can reduce Ag+ ions to silver nanoparticles, which possess excellent antioxidant, antibacterial, and anti-tumor characteristics.

• *C. epitymum* extract could regenerate Ag ions and synthesize silver nanoparticles.
• Morphologically investigated by XRD, FESEM, TEM, and FT-IR, their results showed spherical nanoparticles with a 15–60 nm particle size.
• Silver nanoparticles had significant antioxidant, antibacterial, and cytotoxic properties.

## Graphical abstract

**Figure:**
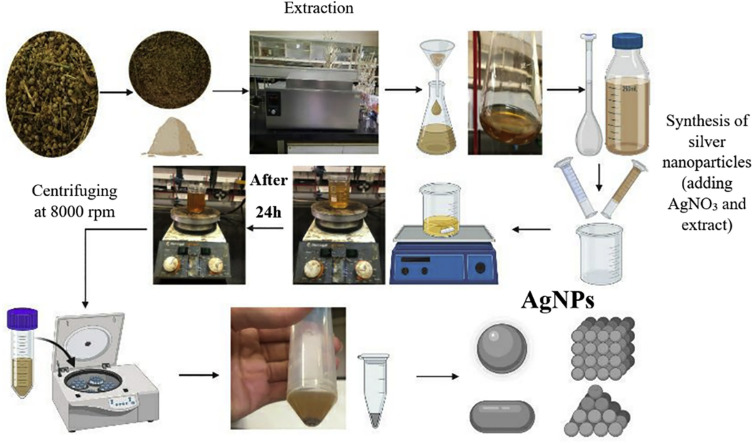

Specifications table

**Table:** 

Subject area:	Chemistry and Phytochemistry and Biological Sciences
More specific subject area:	Green synthesis, Antibacterial properties, Antioxidant properties, Cytotoxic effect
Name of your method:	Green synthesis of silver nanoparticles, evaluation of antibacterial, antioxidant activity, cytotoxic effect on MCF-7 cell line
Name and reference of original method:	Maceration, Green synthesis of nanoparticles, DPPH assay, Disk Diffusion, MTT assay
Resource availability:	Data will be made available on request.

## Background

**Fig. 1:**
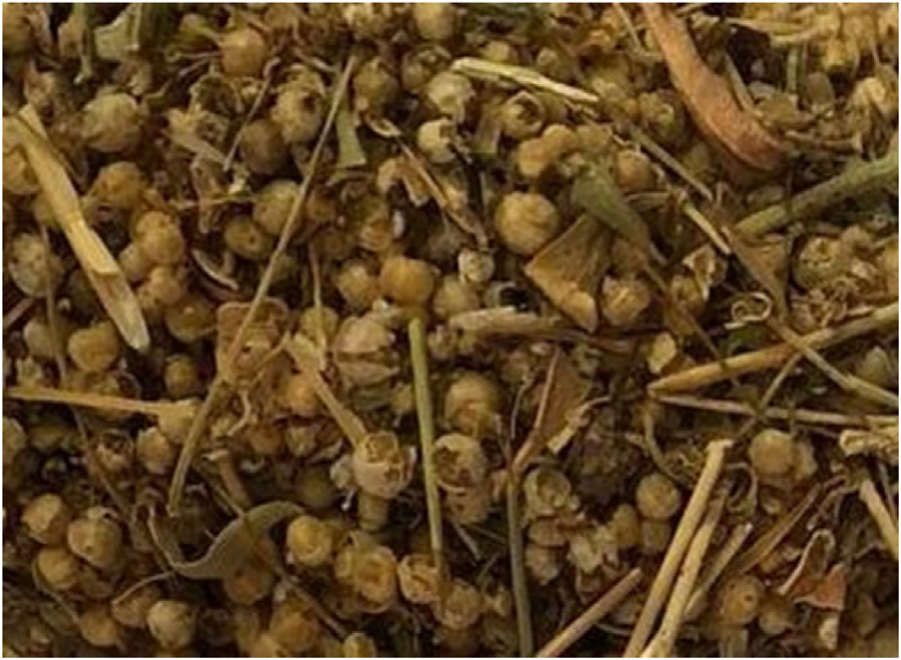
Pictures of *C. epithymum*.

Particles up to 100 nanometers in size are called nanoparticles. Nanotechnology, which involves the production of nanoparticles of different sizes, formats, and compositions, is a sensitive field of investigation with numerous applications. Currently, nanotechnology is growing rapidly due to its widespread application in science and industry [[1], [2]]. Nanoparticle synthesis typically involves physical and chemical methods, but chemical synthesis faces challenges in stability and large-scale production, leading to a demand for environmentally friendly alternatives. Utilizing plants or plant extracts offers a compatible and abundant solution, making the synthesis of noble metal nanoparticles through this method simple and economical [[3], [4], [5]]. The method of green synthesis of metal ions using plant compounds is usually a one-step reaction that transforms into nanoparticles without the need for stabilizing agents. Biologically active substances and compounds found in plant extracts, such as flavonoids and other effective active metabolites soluble in water, can reduce metal ions and form nanoparticles at room temperature [6]. Using this method has the advantage of being non-toxic [5] and applies to fields such as electronics, magnetism, optics, and information storage. Doctors use metal nanoparticles like silver, gold, platinum, and palladium for medical purposes, with silver nanoparticles being widely used for treating diseases due to their potential anticancer, antibacterial, antifungal, and antiviral properties [[7], [8], [9], [10], [11]]. Cancer is a group of diseases where cells lose their ability to grow and divide normally, leading to uncontrolled multiplication. Breast cancer is a type of cancer that causes abnormal growth in breast tissue and is more prevalent in women, but it can also affect men. In recent years, there has been an increased interest in using nanoparticles (NPs) in biosensors to detect and treat cancer, as evidenced by research studies [[12], [13], [14]]. When the production of free radicals exceeds the body's ability to counteract them, it can result in oxidative stress, which increases the risk of developing diseases such as cancer. Antioxidants are substances and procedures that eliminate these harmful molecules or impede their formation. *Cuscuta epithymum*, a medicinal herb belonging to the Convolvulaceae plant family with over 150 species found worldwide [15], has been studied for its antioxidative and immune-boosting effects due to its active compounds such as flavonoids, lignans, quinic acid, and polysaccharides [16]. Fig. 1 shows a view of *C. epithymum. C. epithymum*, also known as the common dodder, produces hundreds to thousands of seeds despite the limited photosynthetic activity and has been used in traditional remedies due to its antimicrobial and cytotoxic effects [17]. This study aims to create AgNPs using an AgNO_3_ solution and *C. epithymum* aqueous extract obtained via the maceration method. confirm the nanoparticles creation through UV–Vis spectroscopy. Their functional groups by FT-IR and morphological characters by TEM, FE-SEM, and XRD were determined. The antioxidant properties using the DPPH assay, the antibacterial properties by the Disk Diffusion method, and their cytotoxic effect on MCF-7 via the MTT assay were calculated.

## Method details

### Plant extract preparation

The dehydrated form of the plant was acquired. A maceration technique was employed for extraction. A precise scale was used to measure 5 g of *C. epithymum* powder, which was then carefully deposited in a sterilized screw bottle. For the extraction process, 100 mL of distilled water was employed as a solvent for the aqueous extract. The solution was heated in a water bath, maintaining a temperature range of 80–90 °C, for 45 min. The extract solution was filtered using Whatman Filter Paper No. 2. Finally, the extracted solution was transferred to a suitable container and stored at the appropriate temperature until further usage.

### Synthesis of silver nanoparticles

To create silver nanoparticles, a mixture of 75 ml of the extract and 25 ml of silver nitrate (AgNO_3_) with a concentration of 2 mM from the German company Merck was prepared. The mixture was then left in the dark at a temperature of 25 °C overnight to allow the reduction of silver ions. The solution's color transitioned from a pale yellow to a rich brown or black hue, signifying the successful creation of silver nanoparticles. Subsequently, the nanoparticle-containing solution was subjected to centrifugation at a rate of 8000 rpm for 20 min. The liquid above the sediment was decanted, and the remaining sediment was dried. The resulting silver nanoparticles were then preserved in a microtube at a temperature of 4 °C for subsequent analysis.

### Characterization of AgNPs

#### Identification of AgNPs using ultraviolet-visible spectroscopy

UV–Vis spectrophotometry (using a PG instrument two-beam) was utilized to scan for the absorption peak between 350–700 nm to verify the existence of silver nanoparticles, as silver tends to absorb within this range.

#### X-ray diffraction (XRD)

The crystallography and dimensions of the synthesized nanoparticles were determined using XRD analysis. The XRD instrument used for this purpose was a copper-based one located at the Plasma Physics Complex in the Science and Research Branch of Tehran's Islamic Azad University.

#### Field emission scanning electron microscope (FESEM)

The Xmu-Mira FESEM was used to analyze the morphology of the synthesized silver nanoparticles.

#### Transient electron microscope (TEM)

Using the 912AB-LEO model electron microscope, the size and dispersion distribution of the silver nanoparticles were established. The electron microscope emitted an electron beam at 120 kV applied voltage.

#### Infrared Fourier transform spectroscopy (FTIR)

The functional groups of AgNPs were identified using FTIR within the 400–4000 cm^-1^ range.

#### Evaluation of antioxidant properties by free radical scavenging (DPPH)

The antioxidant properties of AgNPs were evaluated by testing them with a DPPH solution of 0.004 % concentration. The nanoparticles were mixed with the solution at various concentrations (31.25, 62.5, 125, 250, and 500 mg/L) and incubated in the dark for 30 min. The absorbance at 517 nm was measured using UV–vis spectroscopy. Ascorbic acid was used as a reference antioxidant. The scavenging activity of the free radicals was determined using a specific formula:

$$\%RSC=\left(A_{0}-A_{1}\right)/A_{0}\times 100$$

$$A_{0}=DPPHAbsorbance,A_{1}=SampleorStandardAbsorbance$$

Each sample's antioxidant activity was measured using the IC_50_ value, which represents the concentration needed to inhibit 50 % of DPPH radical formation. The inhibition curve was used to determine this value.

### Antibacterial assay

#### Disk diffusion method

The agar disk diffusion method was used to confirm the antibacterial properties of the silver nanoparticles that were generated. A nutrient agar medium was prepared and sterilized before being poured into Petri dishes. Each dish was then given 10 ml of culture medium. Once the medium had solidified, the bacteria of interest were cultured on the nutrient agar plates. Sterilized 6 mm discs (Pad tan Teb) were loaded with varying concentrations (10, 20, 40, 80 ppm) of the synthesized silver nanoparticles, autoclaved, and placed on the agar plates. The plates were 24 hours thenubated at 37 °C for 24 hours, and the diameter of the growth inhibition zone was subsequently measured in millimeters [18].

### Cytotoxic effect

#### MTT assay

Breast cancer MCF-7 cells were cultured in RPMI 10 % culture medium. The cells were taken out from the CO_2_ incubator, and the supernatant was removed. The cells were then transferred to a flask containing 5 cc of culture medium and passaged into fresh medium and new flasks at specific time intervals. An inverted microscope was used to detect any signs of contamination and determine the number of viable cells. The cells were detached from the bottom of the plate using trypsin enzyme and counted using trypan blue solution. They were then transferred to a 96-well plate for the MTT test. The cells were treated with synthesized silver nanoparticles using the C. epithymum plant extract at various concentrations for 24 hours. The experiment was repeated three times for each concentration of nanoparticles and incubation time. Following this, a formazan solvent was added, and the color intensity was measured using an ELISA microplate reader at a wavelength of 570 nm. The percentage of viable cells for each nanoparticle concentration was calculated using the bellow of formula below [19]:

$$\%\;Viability\;=\;\frac{Average\;optical\;absorbance\;for\;each\;concentration}{Mean\;optical\;absorbance\;of\;the\;control}\;\times \;100$$

## Method validation

**Fig. 2:**
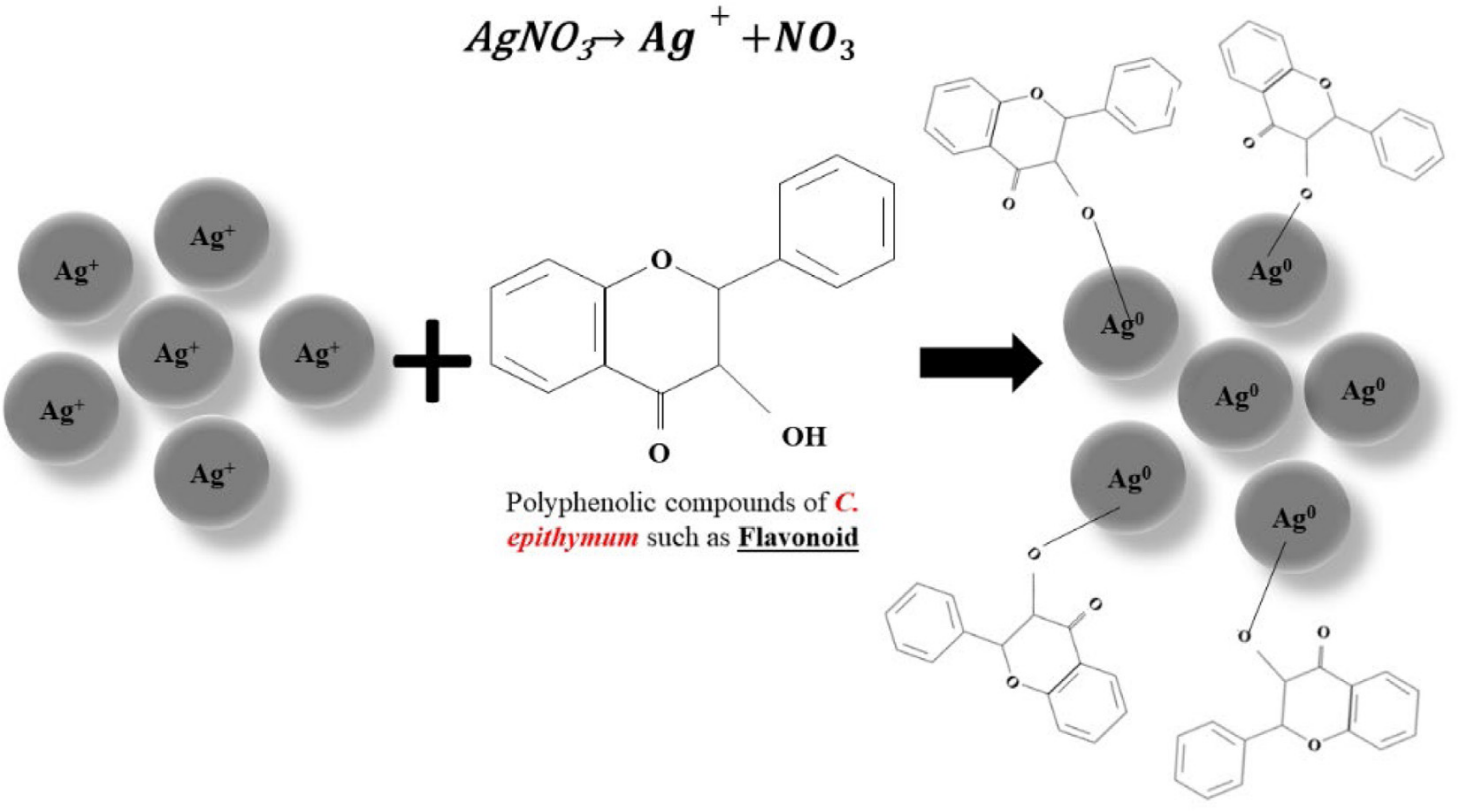
The possible mechanism for green synthesis of Ag-NPs by using *C. epithymum* extract.

**Fig. 3:**
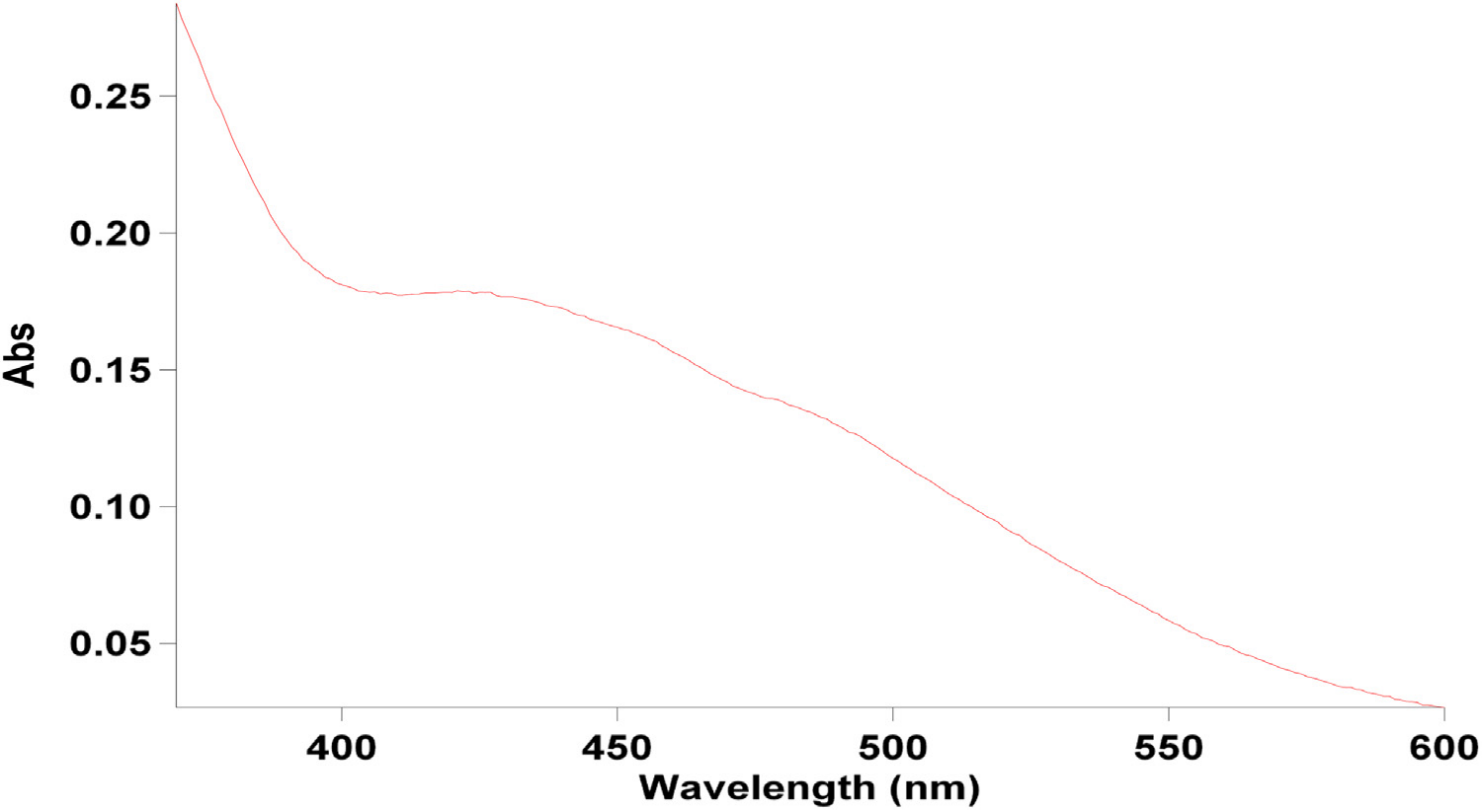
The successful formation of silver nanoparticles was confirmed by the absorption peak observed at 425 nm in the UV-visible spectrum.

The utilization of silver nanoparticles is widespread, but the chemical methods employed for their synthesis often result in environmental issues. To address the issue of environmental pollution caused by physical and chemical synthesis methods, scientists have investigated the use of extracts from medicinal plants for the green synthesis of silver nanoparticles. These nanoparticles can bind to biological molecules and disrupt inhibitory components in bacteria, leading to their demise [[20], [21], [22]]. While the presence of silver ions can result in oxidative stress, some plants naturally contain phenols and flavonoids, which possess antimicrobial and antioxidant properties that can prevent cellular damage. Certain plants can convert Ag^+^ ions to Ag^0^ through bioremediation and produce nanoparticles with antioxidant properties [[23], [24]]. *C. epithymum* due to having phytochemical compounds such as flavonoids, lignans, quinic acid, and polysaccharides as reducing agents and singlet oxygen scavengers, facilitated the reduction of metal salt of silver nitrate to nanoscale by donating electrons to convert Ag^+^ ions to Ag^0^ ions. The possible mechanism for the green synthesis of Ag-NPs is shown in Fig. 2. Over time, the solution turns dark brown, with the final color depending on the duration. The emergence of silver nanoparticles and the stimulation of surface plasmon vibrations are indicated by the brown color. Fig. 3 depicts the findings obtained from UV–Vis spectroscopy. The existence of AgNPs can be deduced from the absorption peak observed at a wavelength of 425 nm [[25], [26], [27], [28], [29]].

### Structural phase and particle size analysis

**Fig. 4:**
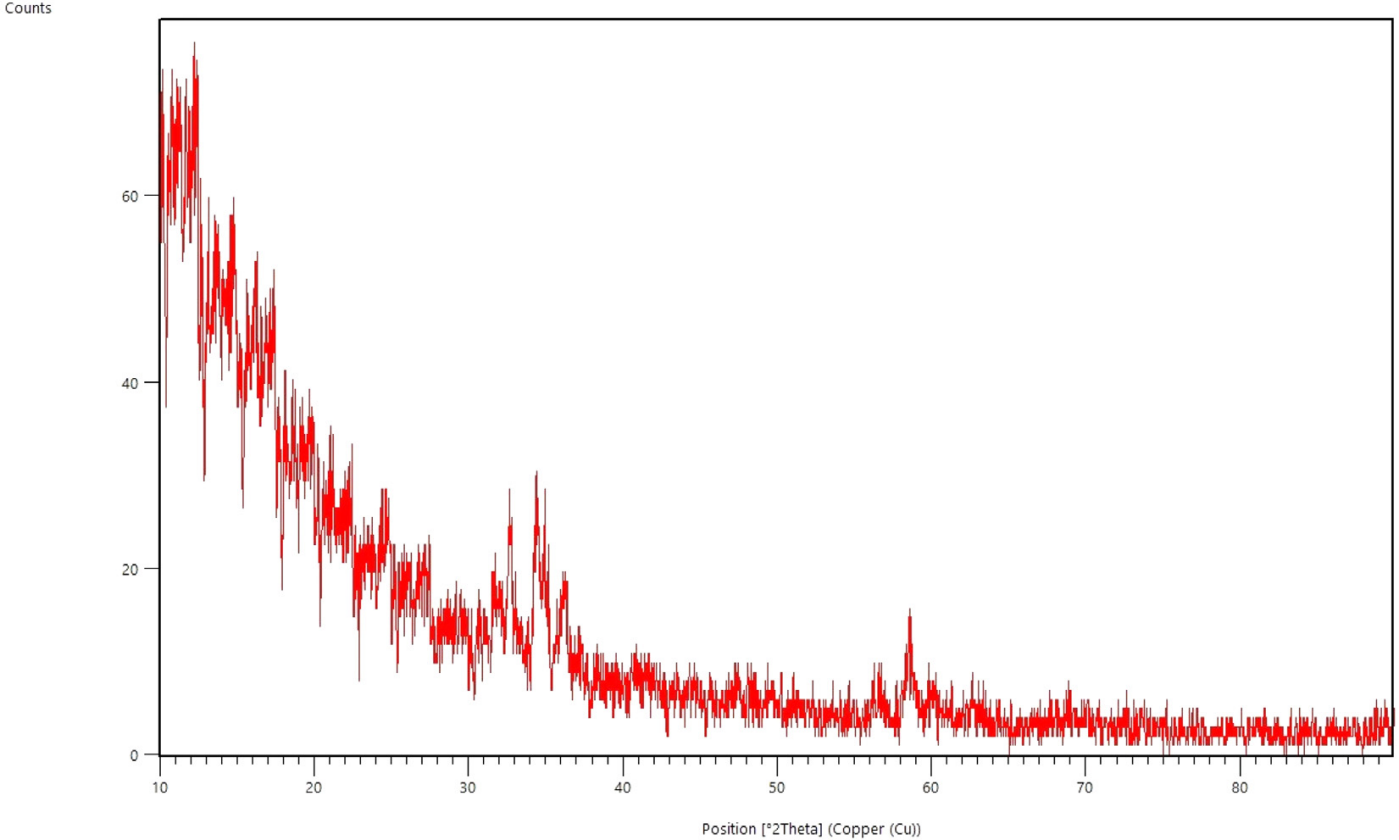
Analysis of the X-ray diffraction spectrum (XRD): It was found that the synthesized silver nanoparticles have high purity and are crystallized, as evidenced by the presence of strong and distinct peaks.

Fig. 4 shows the X-ray diffraction pattern of silver nanoparticles synthesized by *C. epithymum*. The pattern indicates that the nanoparticles are spherical, with peaks at 2θ values of 27.84°, 32.23°, and 46.18° Furthermore, the XRD pattern corresponds to the reference pattern (JCPDS No. 04–0783) for silver nanoparticles. The provided formula can be utilized to calculate the crystallite size of these nanoparticles:

$$D=\;\frac{k\lambda }{\beta cos\theta }$$

Using a form factor of 0.9, an X-ray wavelength of 1.5406 ^Å^ (λ), and the diffraction angle (θ), the given equation yields a full width at half maximum peak diffraction (β). Based on these values, it is estimated that the size of AgNPs is approximately 29 nm.

### Chemical components

**Fig. 5:**
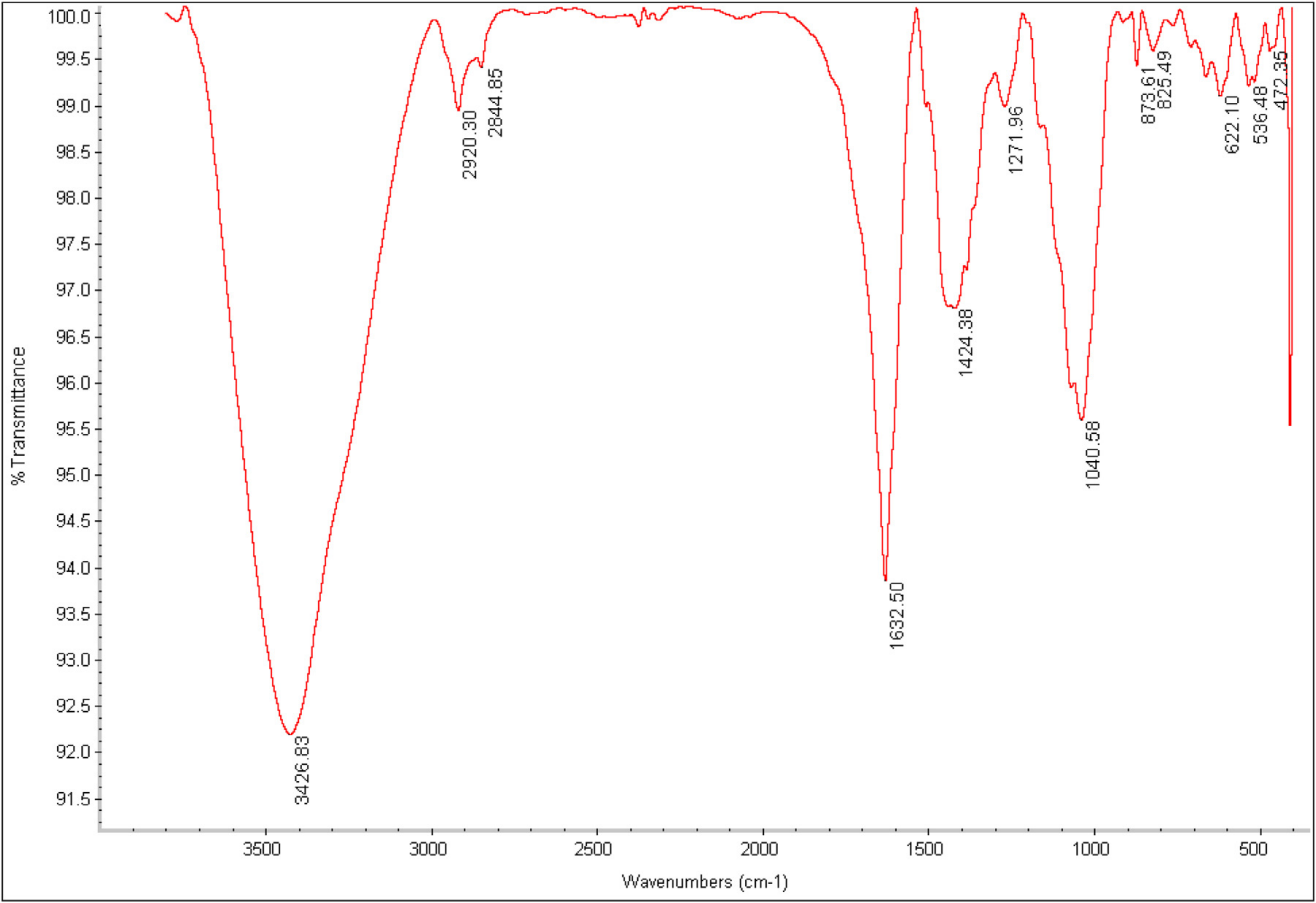
FT-IR spectrum of synthesized Ag nanoparticles. As shown, the intensity of most bands decreases due to a decline in Ag ions.

The nanoparticles' surface was found to have various functional groups through FT-IR analysis, such as hydroxyl, carboxyl, and carbonyl, which contributed to their negative charge. Fig. 5 depicts this. The *C* = *C* stretching vibration of aromatic rings is represented by the peak at 1632.50 cm^-1^, and the C–H bending is represented by the peak at 1424.38 cm^-1^. The C—O tensile vibration bonds in the ester and acid groups are related to the peaks observed at 1040.58 cm^-1^, while the bonds at 2844/85 and 2920/30 cm^-1^ correspond to the C—H tensile bond in alkane compounds. The strong peaks at 3426.83 cm^-1^ are due to -OH tensile, which is caused by the presence of phenolic compounds in the *C. epithymum* extract that resulted in the reduction of silver ions during the synthesis of silver nanoparticles.

### Morphological analysis

**Fig. 6:**
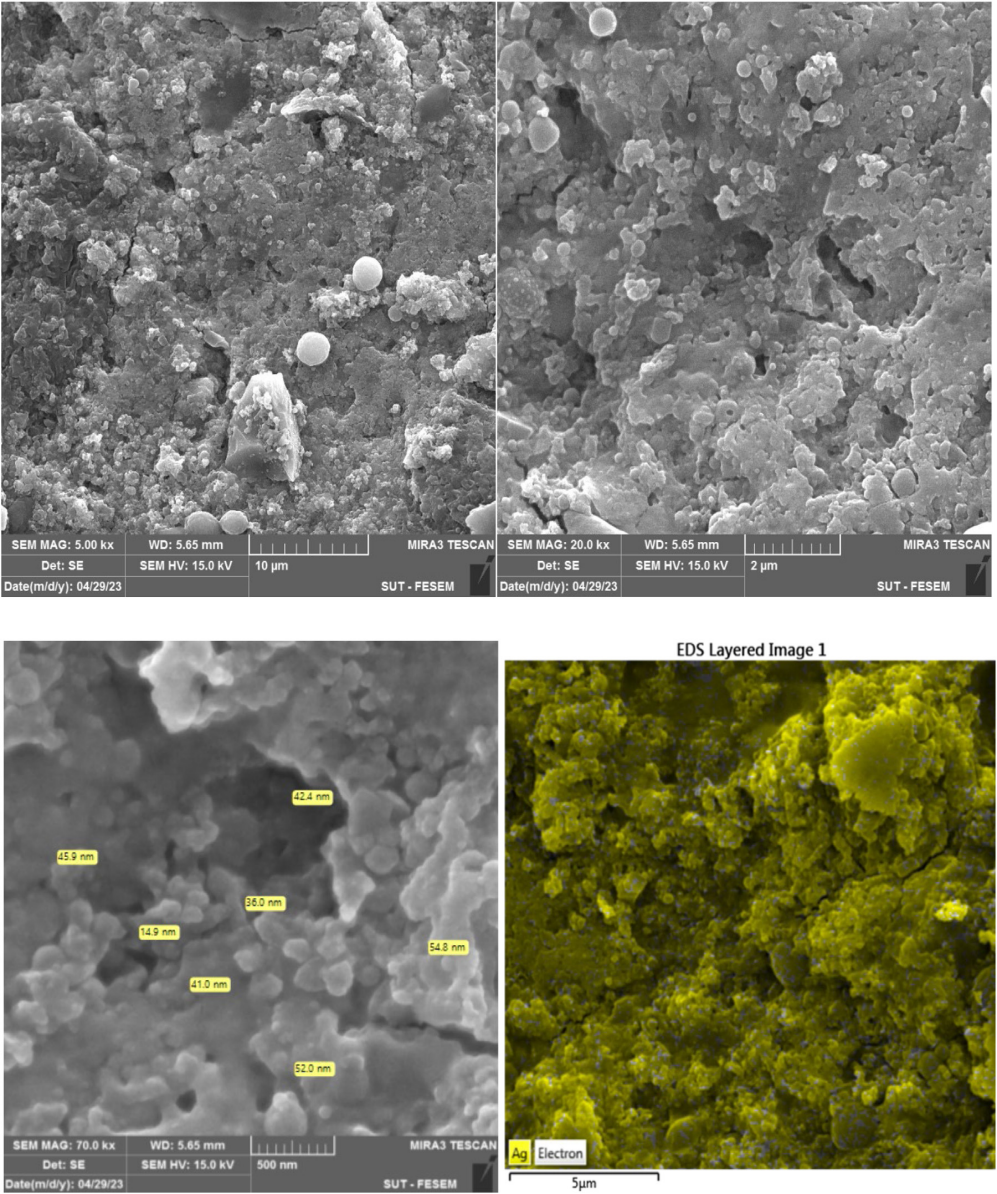
FESEM image and EDS map of Silver Nanoparticles Synthesized with *C. epithymum* extract at various magnifications. The majority of Ag nanoparticles exhibited a spherical shape, with sizes ranging from 15 to 60 nm.

The spherical silver nanoparticles observed in Fig. 6 using FESEM imaging ranged from 15 to 60 nm in size. Also, the elemental mapping results indicated the maximum distribution of silver, suggesting that silver was the predominant element in the respective product [21,30].

**Fig. 7:**
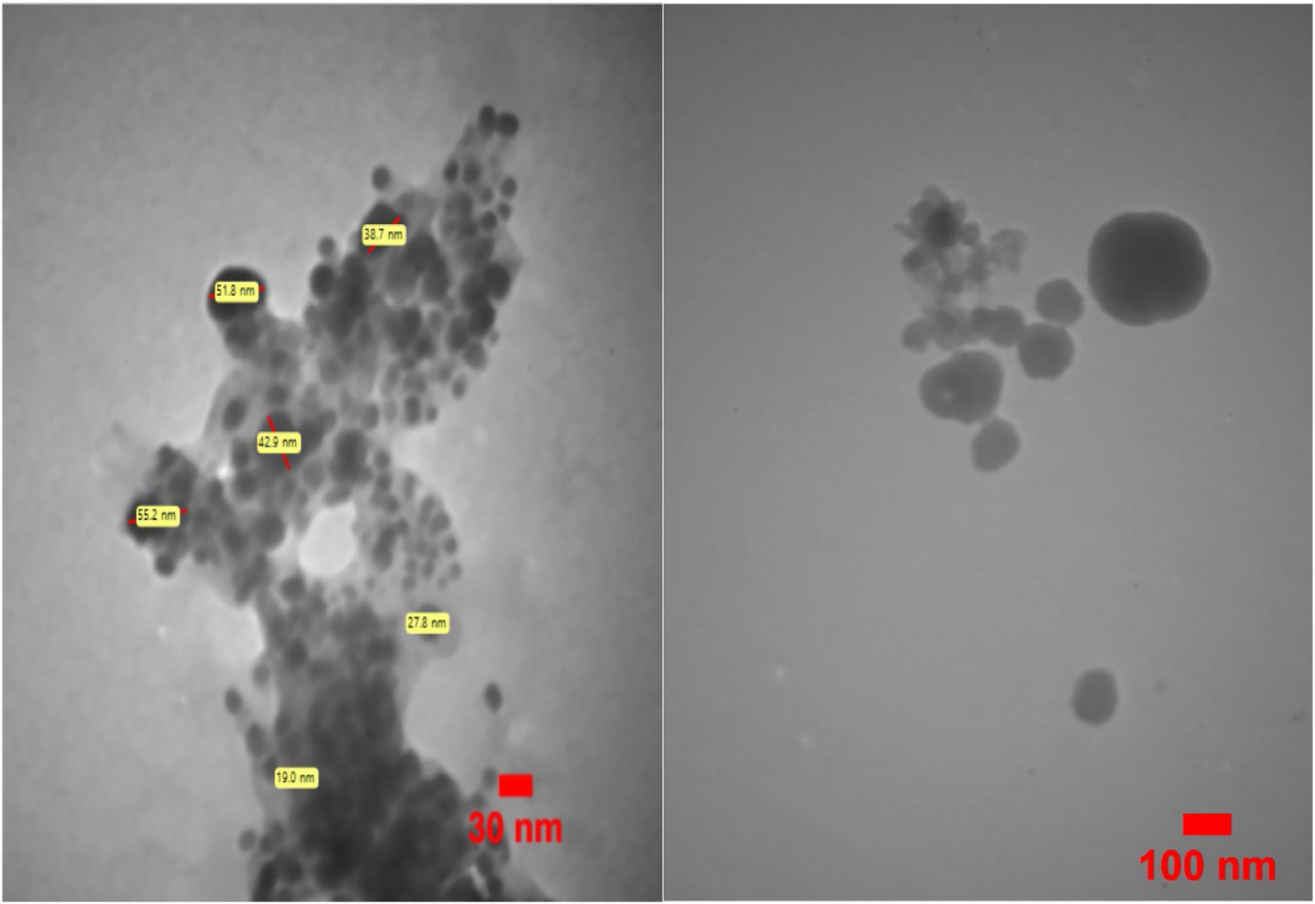
TEM image of silver nanoparticles synthesized with *C. epithymum* extract. The Ag nanoparticles exhibited a spherical shape.

The transmission electron microscope TEM image in Fig. 7 displays the AgNPs that were produced using *C. epithymum* extract. The images show that the silver nanoparticles have a spherical shape and are evenly distributed around the nanoparticles. The background appears lighter, which is due to the solvent used. This is because the density of the solvent is lower than that of the silver nanoparticles, making the nanoparticles appear darker and the solvent appear lighter in the image.

### Antioxidant activities

**Fig. 8:**
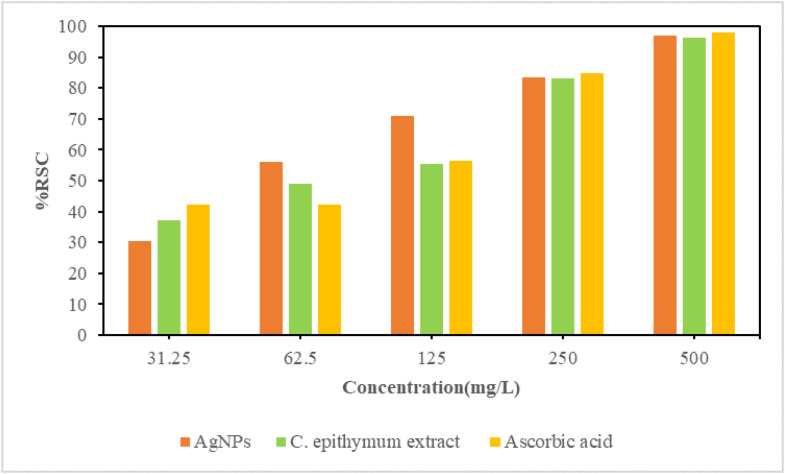
The DPPH free radical scavenging activity was evaluated using nanoparticles synthesized by the hydroalcoholic extract of *C. epithymum* extract. The results were compared to a positive control of standard ascorbic acid at various concentrations.

Furthermore, the study explored the antioxidant characteristics of the nanoparticles, acknowledging the significance of uncovering natural and synthetic antioxidants to counteract oxidative stress and its detrimental effects. The results demonstrated that the nanoparticles effectively eliminate 2,2-diphenyl-1-picrylhydrazyl free radicals by accepting electrons or free radicals and stabilizing hydrogen molecules. *C. epithymum* plant possesses antioxidant properties and can transfer hydrogen to oxidants. The antioxidant activity of the synthesized nanoparticles was found to be concentration-dependent, with higher concentrations resulting in greater antioxidant activity. The antioxidant properties of silver nanoparticles are demonstrated in Fig. 8 and Table 1. The results showed that the synthesized nanoparticles had an IC_50_=45.55 mg/L for 50 % free radical scavenging, which was lower than the IC_50_ =77.94 mg/L and the standard antioxidant ascorbic acid IC_50_=78.23 mg/L. This indicates that the synthesized nanoparticles exhibited excellent antioxidant activity. Furthermore, the antioxidant activity of AgNPs was found to be stronger than that of the extract and ascorbic acid, suggesting that the reduction of silver ions by the plant extract enhanced the antioxidant activity [31].

**Table 1:** Percentage of free radical scavenging by varying concentrations of nanoparticles synthesized using the aqueous extract of C. epithymum.

Percent of inhibition of extract	Absorbance	Concentration of extract (mg / L)
30.40 %	0.4942	31.25
56.09 %	0.3118	62.5
71.02 %	0.2058	125
83.37 %	0.1181	250
97.16 %	0.0202	500

It can be observed that as the concentration of the silver nanoparticles that were synthesized increased, their rate of inhibition also increased.

### Antibacterial activities

**Fig. 9:**
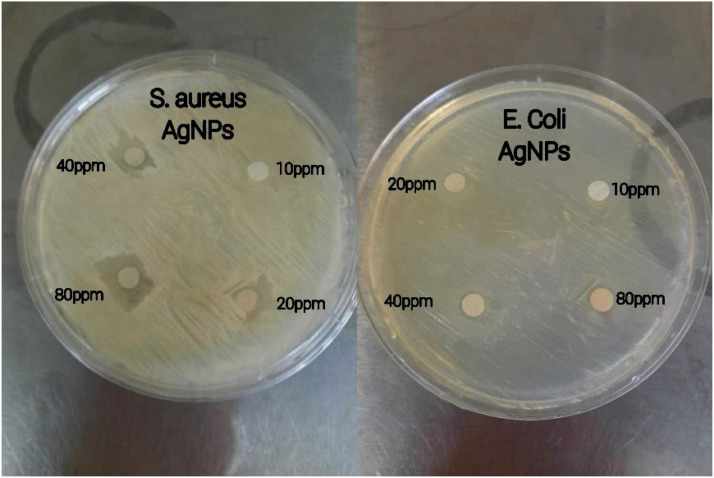
shows that the antibacterial activity of AgNPs was more effective against *E. coli* compared to S. aureus, as indicated by the inhibition area.

According to the findings in Fig. 8 and Table 2, the silver nanoparticles synthesized using *C. epithymum* extract exhibited stronger antibacterial effects against Gram-negative *E. coli* than Gram-positive S. aureus. The largest inhibition zone was observed at 80 ppm nanoparticle concentration, while the smallest was at 20 ppm. This aligns with previous studies and the potent antibacterial properties of silver nanoparticles synthesized from Mentha pulegium plant extracts against *E. coli* and S. aureus [32] (Fig. 9).

**Table 2:** Mean Diameter (mm) of Non-growth Halo of Nanoparticles Synthesized with Aqueous Extract of C. epithymum on Bacteria.

Concentration(ppm)				
Bacteria strains	10ppm	20ppm	40ppm	80ppm

*E. coli*	0	8 mm	9mm	10mm
S. aureus	0	12 mm	14 mm	17 mm

### Cytotoxic effects

**Fig. 10:**
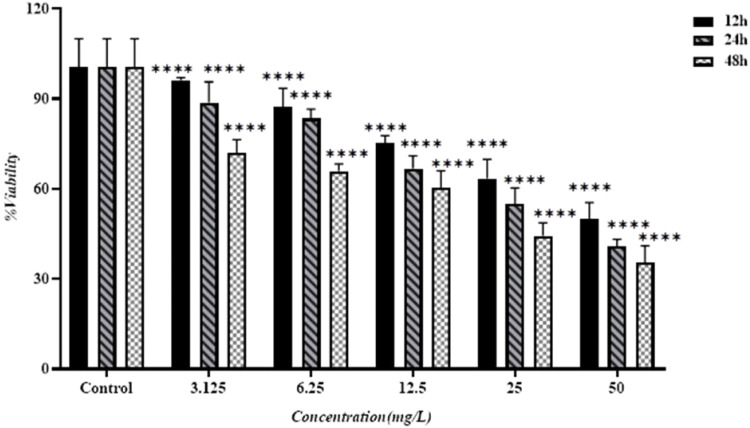
Assessing the effectiveness of synthesized silver nanoparticles on MCF-7 cells at various incubation durations in comparison to the control. The comparison between the three incubation durations and the control revealed a highly significant difference with a P-value < 0.0001.

The cytotoxic effects of silver nanoparticles (AgNPs), synthesized using a green method from *C. epithymum* plant extract, were evaluated. The MTT assay results indicated that cell viability varied based on concentration and time. As the concentration of silver NPs increased, the viability of MCF-7 cells decreased. Fig. 10 illustrates the survival rate of MCF-7 cells treated with silver NPs at different time points. The results showed that the IC_50_ values were 42.53 µg/mL, 36.78 µg/mL, and 26.86 µg/mL after 12, 24, and 48 hours of treatment, respectively [33].

## Limitation

The study indicates that silver nanoparticles from *Cuscuta epithymum* extract exhibit strong antibacterial, antioxidant, and cytotoxic effects on MCF-7 cells due to their bioactive components. Consequently, the sustained release of silver nanoparticles in nano drug delivery systems can effectively deliver breast cancer chemotherapy drugs to the desired location inside the body, their stability may be affected by factors like pH and extract amount. Variations in evaluation methods and plant compounds can influence their activity. Cytotoxicity may occur through necrosis or apoptosis, suggesting that results may be limited. It is recommended to optimize synthesis conditions for reliable results and conduct further tests, including flow cytometry and additional antibacterial and antioxidant assessments.

## Ethics statements

None.

## CRediT authorship contribution statement

**M. Fereydani:** Investigation, Formal analysis, Writing – review & editing, Writing – original draft. **A. Jalalian:** Writing – review & editing, Writing – original draft. **N. Saber:** Investigation, Formal analysis.

## Declaration of competing interest

The authors declare no conflict of interest.

## Data Availability

Data will be made available on request.
